# Correction: Hospital-at-home care in Singapore: A qualitative exploration of health system partners’ state of readiness, and policy and implementation strategies essential to support scale-up

**DOI:** 10.1371/journal.pone.0331758

**Published:** 2025-09-05

**Authors:** Crystal Min Siu Chua, Eward Wei Zheng Lim, Win Hon See Tho, Yuka Asada, Karen E. Peters, Yi Feng Lai

The Acknowledgement section is not indicated. The correct Acknowledgement section is: The authors would like to acknowledge Dr. Stephanie Ko (National University Hospital, Singapore) and Professor Nick Sevdalis (National University of Singapore) for their valuable expert advice on clinical and implementation research methods during the conceptualisation and planning of this study.

The Funding statement for this article is incorrect. The correct Funding statement is as follows: This study received funding support from the National Medical Research Council’s Population Health Research Grant (Reference: PHRGOC23jan-0002), which was used solely for the subscription of Atlas.Ti Web software for data management. The funding body had no involvement in the design, conduct, analysis, or reporting of the study.
