# Supplementary figures and images for: Hospital-at-home care in Singapore: A qualitative exploration of health system partners’ state of readiness, and policy and implementation strategies essential to support scale-up
Source: PLoS One. 2025 Jun 2;20(6):e0323679. doi: 10.1371/journal.pone.0323679 (PMC12129231; doi:10.1371/journal.pone.0323679)

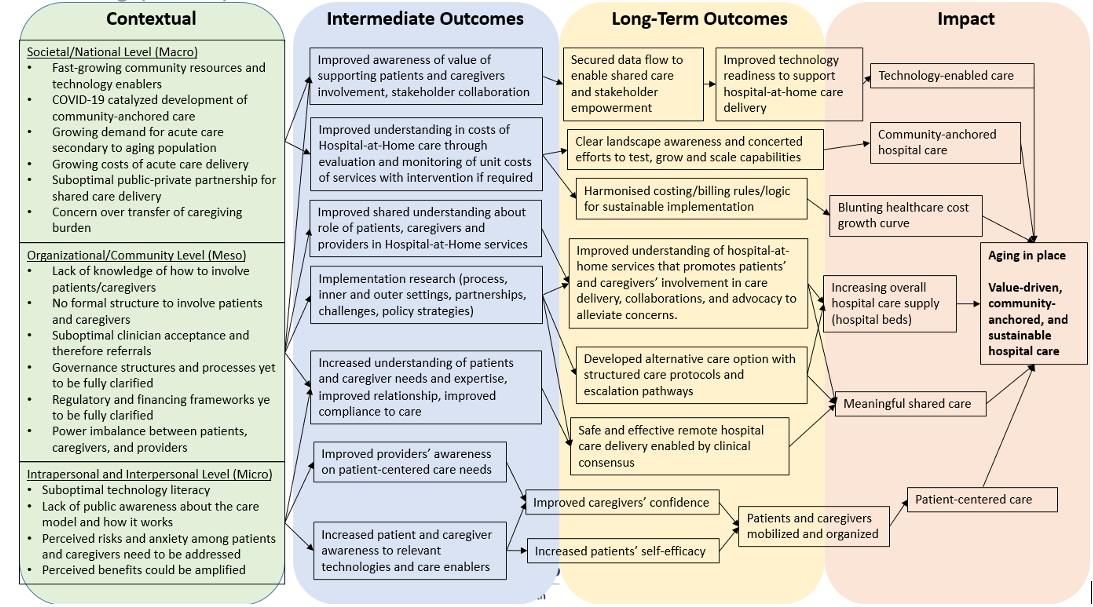

Supplement: S1 — Theory of Change for MIC@Home. (JPG) [file pone.0323679.s001.jpg]
