# Supplementary material for: Hospital-at-home care in Singapore: A qualitative exploration of health system partners’ state of readiness, and policy and implementation strategies essential to support scale-up
Source: PLoS One. 2025 Jun 2;20(6):e0323679. doi: 10.1371/journal.pone.0323679 (PMC12129231; doi:10.1371/journal.pone.0323679)
Supplement: S2 — Interview guide. (DOCX) [file pone.0323679.s002.docx]

**Table S2 Readiness, and Policy and Implementation Strategies Essential to Support Scale-Up Interview Guide**

| **Date of Interview:**   \|  \|  \|  \|  \|  \|  \| \| --- \| --- \| --- \| --- \| --- \| --- \| | | |
| --- | --- | --- | --- | --- | --- | --- | --- | --- |
| **Location:** |  | |
| **Interviewer Name(s):** |  | |
| **Interviewee Name(s):** |  | |
| **Time:** | Start \|__\| | End \|__\| |

*This interview is a component of a research study exploring the Health System Partners’ State of Readiness, and supporting Policy and Implementation Strategies Essential to Support Scaling-Up Efforts of Hospital-at-Home in Singapore.*

*We have selected you because you have had a major role in the initial implementation phase of Hospital-at-Home (HaH) or Mobile Inpatient Care at Home (MIC@Home), as it is known here in Singapore. We are exploring strategies and enablers to facilitate the shift from pilot/sandbox phase to mainstream.*

*The interview will focus on your perception of the state of readiness of Singapore and key barriers and enablers with regards to mainstreaming and scaling up. We hope that you will respond from your position as a key stakeholder in implementation, and not as a reflection of your organisation’s perspectives.*

*Remember, you can choose not to answer any question that makes you feel uncomfortable for any reason. Your identity will be treated with confidentiality, and any information you've shared during our conversation today will be kept anonymous.*

*This interview should take about 1 hour.*

*Any questions or comments before we begin? [wait for questions]*

*Great. So, let’s get started. I’m going to turn on the recorder now. I’ll let you know when I turn it off at the end of our discussion as well.*

*Turn digital recorders on.*

Opening & Interviewee’s Role

I would like to start by asking you about your work experiences and specific implementation roles in MIC@Home.

Probes:

1. How long have you been involved in Singapore's healthcare sector, and could you provide an overview of your professional experiences within this industry?
2. What is your involvement in MIC@Home?
3. How did your involvement in MIC@Home begin? Has it evolved?

Research Question 1: What is the state of readiness of Singapore’s health system partners to scale-up the “Mobile Inpatient Care at Home- MIC@Home care model?

Sub-Question 1a: In your opinion, what does “scaling-up” of MIC@Home mean to you?

Probes: Volume, institutions, use cases, care settings (home, nursing homes, etc)

Sub-Question 1b: Help me understand your/your team’s/organisation’s capacity for scaling-up MIC@Home.

Probes:

1. <Depending on the role of the participant in the care model’s implementation> Can you describe the current resources or infrastructure needed to scale MIC@Home?

Sub-Question 1c: In your opinion, are there any significant driving forces/motivators from you/your team/your organization for the scale-up to be successful?

Probe:

1. If yes, please elaborate (e.g., Explore micro intrapersonal, micro interpersonal, meso organizational, macro environmental factors). Can you recall any instances or observations that reflect these motivators?
2. If no, what are the reason(s) that discourages you/people around you from this care model scale-up? Can you recall any instances or observations that reflect this?

Sub-Question 1d: Share your insights on the key system-level facilitators (to have worked well) and barriers to the scale-up of MIC@Home in Singapore.

Probe: Could you provide examples or experiences illustrating these facilitators and barriers? (refer to Table 1 for guide on potential facilitators and barriers)

Research Question 2: What are the effective multi-level strategies for scaling-up of MIC@Home in Singapore?

Sub-Question 2a: In your opinion, what do you think is needed to successfully scale up MIC@Home?

Questions to ask:

- *From your perspective, what national system enablers would be required for Singapore to mainstream the MIC@Home care model? (What are the enablers that needs to happen to MIC meaningfully scale up and mainstream).*
  - *Probe: IT, Logistics*
- *From your perspective, what human resourcing enablers would be required for Singapore to mainstream the MIC@Home care model?*
  - *Probe: education, training. For cluster people, specific to their professional group.*
- *In your opinion, what changes in infrastructure is needed to mainstream MIC@Home care model?*
  - *Probe: service sites, record system, dissemination organization*

Sub-Question 2b: From your perspective, what policy strategies would best prepare Singapore's healthcare system for the scaling-up of the MIC@Home care model?

Questions to ask:

- *From your perspective, what policy strategies would best prepare Singapore's healthcare system to mainstream the MIC@Home care model?*
  - *Probe: Finance, Governance*
- *In your opinion, what kind of partnership/stakeholder relationship is needed to mainstream MIC@Home care model?*
  - *Probe: Advisory/executive board*

Conclusion:

Is there anything else related to MIC@Home scale-up that we have not discussed but you feel is important?

Your time and insights are truly valuable to this study. Thank you very much for your participation.

**Interview Summary or Debrief Sheet**

**Date and Time:
Location:**

**Recording saved:**

This form may be filled out after each interview to capture initial impressions and consulted during the data collection and analysis processes.

**---------------------------------------------------------------------------------------------------------------------**

1. **The main issues or themes that struck me in this interview:**
2. **Summary of information I got (or failed to get) on each of the target questions:**
3. **Anything else that was salient, interesting, illuminating, or important in this interview:**
4. **New (or remaining) target questions I have in considering my next interview:**

**Other resources and definitions**

Definition of “scale-up”:

***Scaling-up****- the process by which health interventions shown to be efficacious on a small scale and or under controlled conditions are expanded under real world conditions into broader policy or practice (WHO, 2010). The concept of scaling up is different from routine adoption as it involves an explicit intent to expand the reach of an intervention to new settings or target groups and is accompanied by systematic strategy to achieve this objective (Milat, 2014).*

***Horizontal Scale-up****- Horizontal scale-up refers to the expansion of an intervention geographically or to new target populations. This could be through replicating a successful program in new locations, adapting the program to serve different population groups, or expanding the services offered as part of the program. The goal of horizontal scale-up is to broaden the reach of the program to benefit more people. (ExpandNet, 2011; Simmons, 2007)*

***Vertical Scale-up****- Vertical scale-up usually refers to the process of integrating an intervention more deeply into an existing system. This could be through policy changes, budgetary allocation, training of staff, or other activities that make the program a more routine or central part of system operations. Vertical scale-up focuses on institutionalizing the intervention to ensure sustainability and long-term impact. (ExpandNet, 2011; Simmons, 2007)*

**Table 1**: Synthesis of success factors and barriers to scaling up public health interventions in rank order of mentions (Milat, 2015)

| Barriers | - Not adapting intervention approaches to the local context - Intervention costs and other economic factors - Lack of human resources - Resistance to the introduction of new practices due to capacity constraints - Insufficient investment in implementation infrastructure including training, monitoring and evaluation systems - Staff recruitment and staff turnover - Lack of political will - Traditional research funding processes are not flexible enough to support evaluation of scale up - Leadership changes amongst implementation agencies - Poor engagement with stakeholders and thought leaders - Poor role delineation - Maintaining quality and consistency of health interventions at scale |
| --- | --- |
| Facilitators | - Establishing monitoring and evaluation systems - Costing and economic modelling of intervention approaches - Active engagement of a range of implementers and the target community - Tailoring scale-up approach to local context and use of participatory approaches - Systematic use of evidence - Infrastructure to support implementation such as training, delivery systems, technical resources - Strong leadership and champions - Political will - Well-defined scale-up strategy - Strong advocacy - Flexible responses to human resource constraints - Formative research to ensure appropriate intervention design - Equity of intervention delivery and monitoring intended and unintended consequences across socio-demographic profiles - Effective communication strategy - Effective governance and coordination - Clear role definition and delineation - Keeping the intervention model simple - Financing models - Programmes are visible, publicized and effectively packaged - Developing strategies for integration into existing services |

Reference:

1. Milat, A.J., Bauman, A. & Redman, S. Narrative review of models and success factors for scaling up public health interventions. Implementation Sci 10, 113 (2015). <https://doi.org/10.1186/s13012-015-0301-6>
2. ExpandNet and WHO. (2011). Nine steps for developing a scaling-up strategy. Geneva: World Health Organization.
3. Milat AJ, King L, Wolfenden L, Rissel C, Newson R, Bauman AE, et al. Increasing the scale and adoption of population health interventions: experiences and perspectives of policy makers, practitioners and researchers. Health Res Policy Syst. 2014, 12(18).
4. Simmons, R., Fajans, P., & Ghiron, L. (Eds.). (2007). Scaling up health service delivery: from pilot innovations to policies and programmes. Geneva: World Health Organization.
5. World Health Organization. ExpandNet: nine steps for developing a scaling-up strategy. Geneva: WHO; 2010.
