# Supplementary material for: Hospital-at-home care in Singapore: A qualitative exploration of health system partners’ state of readiness, and policy and implementation strategies essential to support scale-up
Source: PLoS One. 2025 Jun 2;20(6):e0323679. doi: 10.1371/journal.pone.0323679 (PMC12129231; doi:10.1371/journal.pone.0323679)
Supplement: Attachment — Submitted filename: Response to reviewer_130325.docx [file pone.0323679.s006.docx]

**Response to reviewer**

Title: Hospital-at-Home Care in Singapore: A Qualitative Exploration of Health System Partners’ State of Readiness, and Policy and Implementation Strategies Essential to Support Scale-Up (PONE-D-24-50121)

Revised manuscript by Apr 11 2025

| **Comments by Editor** | **Response to Editor** |
| --- | --- |
| Dear authors,  Thanks for your revision. The reviewers have advised acceptance, but I still have a few comments: 1. The abstract needs to be re-written. The framework used should be in the Background/Methods part, and not in the Results. This should be fixed. | Dear Editor, thank you for reviewing our manuscript, and your comments.  We have reorganized the abstract accordingly on page 2. |
| 2. A brief description of the framework used should be put in the Introduction, also explaining why that particular framework was used. Also, please improve the integration of the framework with the rest of the article. As it stands, the framework does not appear to be well-integrated with the methods and results. The framework should in some sense, guide the questions asked, the analysis, and the presentation of the results. | Dear Editor, thank you for your feedback.  We have added some context about the framework on pages 5 and 6.  Furthermore, we have integrated the framework more thoroughly within the Methods section (page 9) and the Results section (pages 12, 13, 17, and 27). This ensures consistency with its use in guiding the Discussion segment, as previously outlined.  With this addition, the framework now would have been integrated to guide our research questions, analysis in methods, and presentation of results.  Thank you. |
